# Defining the role of *ab externo* Xen gel stent in glaucomatous eyes with prior failed surgical intervention

**DOI:** 10.1007/s00417-022-05857-6

**Published:** 2022-10-22

**Authors:** Lillian K. To, Rupak K. Dhoot, Alice Z. Chuang, Sam Karimaghaei, Francisco Guevara-Abadia, Ruchi D. Shah, Robert M. Feldman

**Affiliations:** 1 Ruiz Department of Ophthalmology and Visual Science, McGovern Medical School at The University of Texas Health Science Center at Houston, Houston, TX USA; 2 Robert Cizik Eye Clinic, 6400 Fannin St., Suite 1800, Houston, TX 77030 USA; 3 Cole Eye Institute, Cleveland Clinic, Cleveland, OH USA

**Keywords:** Xen, Glaucoma, Stent, Glaucoma drainage device, Cyclophotocoagulation, Tube shunt, Trabeculectomy

## Abstract

**Purpose:**

To evaluate the safety and efficacy of Xen45 Gel stent (Xen; Allergan) in eyes that have failed prior surgical intervention, compared to traditional glaucoma drainage device (GDD) or continuous-wave cyclophotocoagulation (CPC). Since this population has low expected success rates with additional surgery, it is vital to compare to standard-of-care surgical options.

**Methods:**

Retrospective, single-center, case–control study of *ab externo* transconjunctival Xen shunt in eyes that have previously undergone trabeculectomy and/or GDD surgery. Postoperative data were collected for 18 months. Failure was defined as no light perception, additional glaucoma surgery required, or intraocular pressure (IOP) of < 6 mmHg after 6 weeks postoperatively.

**Results:**

Eighteen Xen eyes and 36 control eyes matched on both glaucoma type and previous glaucoma surgeries were included. Seventy-two percent had primary open angle glaucoma, 11% uveitic, 6% primary angle closure, 6% pseudoexfoliation, and 6% pigmentary glaucoma. Fifty-six percent of eyes in each group had prior trabeculectomy, 28% of Xen and 31% of control eyes had prior GDD, and 17% of Xen and 14% of control eyes had both. Baseline medicated IOP was lower in the Xen group (21.8 ± 7.2) compared to controls (27.5 ± 9.4, *P* = 0.043). The cumulative failure rate at year 1 was 17% for Xen and 20% for controls (*P* = 0.57). Mean survival time was 14.1 (± 1.5) months and 11.4 (± 0.6) months for controls. There was no difference in minor complication rates between groups (*P* = 0.65), but the Xen group had a significantly lower rate of serious complications (*P* = 0.043) defined as vision threatening or requiring surgical intervention in the operating room. When censored for additional glaucoma procedures, there were no differences at year 1 in IOP, change in IOP, number of IOP-lowering medications, or number of medications reduced from baseline.

**Conclusions:**

The Xen shunt provides a reasonable alternative to current standard of care, with a similar failure rate at year 1, with a noninferior IOP reduction compared to GDD and CPC, and a preferred safety profile.

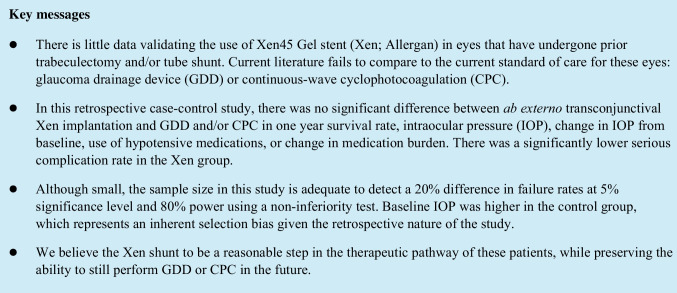



## Introduction

Glaucoma remains a leading cause of blindness worldwide. An estimated 76.0 million people were affected by glaucoma in 2020, with 111.8 million people projected to be affected by 2040 [1]. Surgical treatment options have increased since the introduction of microinvasive glaucoma surgery (MIGS) [2, 3]. These newer surgical options are aimed at lowering intraocular pressure (IOP) with less risk than traditional glaucoma filtering surgery. Most MIGS are designed to increase aqueous outflow, either by utilizing the natural outflow pathway or creating a new outflow pathway.

The Xen45 gel stent (Xen; Allergan) creates a new aqueous outflow pathway that drains into the subconjunctival space, similar to a traditional trabeculectomy. The stent is 6 mm long with a 45-μm lumen and is designed to limit early postoperative hypotony, a significant risk of traditional filtering surgery. The procedure requires use of mitomycin-C (MMC) to reduce postoperative fibrosis [4, 5]. The Xen stent is approved by the US Food and Drug Administration for *ab interno* placement, but in an effort to minimize invasiveness, optimize efficacy, and improve safety as well as ease implantation, an *ab externo* approach has been developed and adopted by many surgeons. This approach can involve an open or closed conjunctiva method [6, 7]. Similar efficacy has been shown using *ab externo* open conjunctiva, *ab externo* closed conjunctiva (or transconjunctival), and *ab interno* approaches, with slight differences in postoperative bleb management [8, 9].

The Xen stent has demonstrated efficacy in IOP reduction in patients with glaucoma refractory to medical management, with 1-year success rates ranging from 15 to 66% [10–12]. However, there is limited published data directly addressing Xen stent success in patients who have failed prior traditional glaucoma surgical interventions. These patients have high failure risks for any surgery involving transconjunctival filtration and may require multiple surgeries. Lewczuk et al. showed similar success rates of Xen stent implantations in surgically naive eyes and eyes with prior glaucoma surgery, with a reoperation rate of 23.9% over 2 years [13]. Hengerer et al. also showed similar success rates for Xen stent implantation in eyes with prior surgery compared to surgically naive eyes but used an *ab interno* approach [14]. In addition, several small case series of Xen implantation after failed trabeculectomy have been published [15, 16]. Most recently, Gallardo et al. compared *ab interno* with transconjunctival *ab externo* Xen placement and included eyes with prior conjunctival incision surgery in their analysis, and found no significant difference in mean IOP or percent reduction between the techniques [17]. However, none of these studies compare the Xen stent with the current standard of care: glaucoma drainage device (GDD) implantation or continuous-wave cyclophotocoagulation (CPC).

To the best of our knowledge, there have not been any published reports regarding the efficacy of the Xen stent implanted via an *ab externo* approach compared to traditional surgery in surgically refractory glaucoma patients (PubMed search July 15, 2022 using terms Xen and glaucoma). Since this population has low expected success rates with any additional surgery, it is vital to compare Xen stent implantation to current standard-of-care surgical options. Our study evaluates the safety and efficacy of *ab externo* Xen stent implantation compared to GDD or CPC in eyes that had previously undergone trabeculectomy or GDD implantation but required further IOP lowering for disease management.

## Methods

This retrospective case–control study was conducted at the Ruiz Department of Ophthalmology and Visual Science at the McGovern Medical School at The University of Texas Health Science Center in Houston (UTHealth) and Robert Cizik Eye Clinic. Institutional Review Board approval was obtained from the UTHealth Committee for the Protection of Human Subjects. All research adhered to the Declaration of Helsinki and was compliant with the Health Insurance Portability and Accountability Act. Data collection conformed with all relevant laws. The Institutional Review Board determined informed consent could be waived for this study.

### Participants

All eyes in which Xen stents were implanted via *ab externo *approach from November 22, 2016 to November 4, 2020 were identified by current procedural terminology (CPT) code 66183 (insertion of anterior segment aqueous drainage device, without extraocular reservoir, external approach) and reviewed. Cases were defined as eyes with surgically refractory glaucoma (uncontrolled IOP after a trabeculectomy or GDD implantation) who underwent Xen stent implantation via *ab externo* approach. 

Each Xen stent case was matched to 2 controls, which were identified by CPT codes for trabeculectomy (66172), CPC (66710), and GDD (66180). Controls were defined as eyes with surgically refractory glaucoma who received an additional filtering procedure (trabeculectomy or GDD) or continuous wave CPC. Controls were matched to cases based on type of glaucoma and type of initial glaucoma filtering surgery. Eyes were excluded if they underwent CPC prior to the study procedure or had less than 3 months of follow-up. If both eyes met eligibility criteria, both eyes were included.

### Surgical technique and postoperative management

All Xen stent implantations were performed using a closed conjunctiva, *ab externo* approach [7, 8, 17]. A temporal paracentesis was made, through which intracameral lidocaine was administered. A superior limbal bridle suture was placed through clear to infraduct the eye. A caliper was used to mark 3- and 7-mm posterior to the superior limbus. The needle of the Xen gel stent injector was tunneled subconjunctivally, entering at the 7-mm mark to the 3-mm mark, where it was then used to create a scleral tunnel into the anterior chamber. Once the needle was seen in the anterior chamber, the stent was deployed and the injector removed. The placement of the stent was then verified, and gentle cautery was applied at the needle entry site. Bleb formation was verified superiorly and any necessary gentle adjustments to the Xen placement were maded before MMC was injected subconjunctivally posterior to the Xen stent at a concentration of 0.2–0.8 mg/mL and a volume of 0.1 mL. The bridle suture was then removed. Patients were instructed to apply topical antibiotics for 1 week. Topical corticosteroids course was identical to routine post-trabeculectomy care, beginning with 4 times a day for 3 weeks, followed by a taper based on bleb appearance.

No medication washout was performed preoperatively. Topical and systemic IOP-lowering medications were suspended on the day of surgery and on postoperative day 1 but were reintroduced at the provider’s discretion. Surgical revisions, such as bleb needling, were performed according to the surgeon’s usual technique.

GDD and continuous wave CPC were performed according to the surgeon’s standard technique at the time of the surgery.

### Data collection

Data were collected for the following patient visits: baseline (last visit prior to surgery), surgery day, and postoperative day 1 (window 1–3 days), week 1 (4–18 days), week 3 (19–45 days), month 3 (46–145 days), month 6 (146–270 days), year 1 (271–540 days), and the last visit (up to 18 months). If there were 2 or more visits within a timeframe, data from the visit closest to the date of interest was used. Data collected at baseline included demographics (age, sex, race, and ethnicity), ocular surgical history, type and stage of glaucoma based on ICD10 code, and most recent visual field mean deviation (MD). At each visit, best-corrected visual acuity (BCVA), IOP, and the number of ocular antihypertensive medications, including systemic carbonic anhydrase inhibitors, were recorded.

Intraoperative procedural details and complications, postoperative complications, surgical revisions, and other surgical interventions were recorded during the follow-up period (up to 18 months) as they occurred. Complications were sorted into two categories: minor and serious. Minor complications included those that were self-limited and non-visually threatening, and did not require surgical intervention or only required intervention in the minor procedure room or office. Serious procedures included any visually threatening compilation or complication requiring reoperation in the operative suite.

### Outcomes

Failure was defined as meeting any of the following conditions in the follow-up period: 1) no light perception (NLP) vision; 2) additional glaucoma surgery required; 3) persistent IOP of less than 6 mm Hg after 6 weeks postoperatively. Revisions, including bleb needling, were not considered surgical failures.

The primary efficacy outcome was the cumulative incidence of failure at 1 year. Success was defined as the reciprocal of failure, also referred to as survival. The safety outcome variables were the incidence and number of complications and revisions and incidence of permanent loss of 2 lines or more of BCVA.

### Sample size calculation

With an assumed overall failure rate for primary failure criteria at 1 year of 5% for controls [18] and a non-inferiority margin of 7.5%, a sample size of 15 cases and 30 controls was required to detect a 20% difference in failure rates at 5% significance level and 80% power using a non-inferiority test.

### Data analysis

Snellen BCVA was converted to logMAR by – log_10_(BCVA), with count figures as 20/1500, hand motion as 20/4000, light perception as 20/8000, and NLP as 20/20,000. At each scheduled visit, the change in IOP, change in number of IOP-lowering medications, and change in BCVA from baseline were calculated.

Continuous variables were summarized by mean (± standard deviation) and range and compared between groups using the two-sample *t* test. Discrete variables were summarized by frequency (%) and compared between groups using the Fisher exact test. Kaplan–Meier survival curves were calculated to estimate cumulative failure rates at 1 year for each group and to compare between groups. Log-linear regression analysis was used to compare the number of complications, revisions, and additional ocular procedures between groups.

All computations were performed using SAS v9.4 for Windows. A *P* value less than 0.05 was considered statistically significant.

## Results

### Demographics and ocular characteristics

A total of 18 case (Xen) eyes and 36 control eyes were included. Mean duration from surgery to last follow-up was 15.8 months (± 3.9; range 1.9–18.0), with an average of 14.3 months follow-up (± 4.4; range 1.9–18.0) for the Xen group and 16.6 months (± 3.4; range 2.6–18) for controls. The majority of eyes were diagnosed with primary open-angle glaucoma (72% for each group) as seen in Table 1. Fifty-six percent of eyes in each group had prior trabeculectomy, 28% of Xen and 31% of control eyes had prior GDD, and 17% of Xen and 14% of control eyes had both. Baseline demographics and ocular characteristics, including visual field damage, were similar between groups, except baseline IOP (medicated) was significantly lower in the Xen group (*P* = 0.043).

**Table 1:** Demographics and baseline characteristics

	Overall (*N* = 54)	Xen (*N* = 18)	Control (*N* = 36)	*P*
Demographics				
Age (years, ± SD, [range])	63.9 (± 13.5) [21–85]	65.4 (± 15.6) [26–85]	63.1 (± 12.5) [21–81]	0.55
Sex (males, %)	34 (63%)	14 (78%)	20 (56%)	0.14
Race/ethnicity (%)				0.60
White	23 (43%)	10 (56%)	13 (36%)	
Black	23 (43%)	6 (33%)	17 (47%)	
Hispanic	7 (13%)	2 (11%)	5 (14%)	
Asian	1 (2%)	0 (0%)	1 (3%)	
Ocular characteristics				
Study eye (right, %)	28 (52%)	7 (39%)	21 (58%)	0.25
Glaucoma type (%)				1.00
Primary open angle	39 (72%)	13 (72%)	26 (72%)	
Primary angle closure	3 (6%)	1 (6%)	2 (6%)	
Pseudoexfoliation	3 (6%)	1 (6%)	2 (6%)	
Pigmentary	3 (6%)	1 (6%)	2 (6%)	
Uveitic	6 (11%)	2 (11%)	4 (11%)	
Visual field (MD, ± SD, [range])	− 16.4 (± 10.1) [− 31.3 to − 1.2]	− 15.4 (± 9.8) [− 31.3 to − 1.2]	− 17.0 (± 10.4) [− 31.1 to − 2.3]	0.64
Stage of glaucoma (%)				0.32
Moderate	13 (24%)	6 (33%)	7 (19%)	
Severe	41 (76%)	12 (67%)	29 (81%)	
Intraocular pressure (mmHg, ± SD, [range])	25.6 (± 9.0) [12–56]	21.8 (± 7.2) [12–35]	27.1 (± 9.5) [16–56]	0.043
Number of IOP-lowering medications (*n*, ± SD, [range])	2.6 (± 1.1) [0–5]	2.3 (± 1.2) [0–4]	2.8 (± 1.1) [0–5]	0.093
Best corrected visual acuity (logMAR, ± SD, [range])	0.46 (± 0.67) [0.0–2.3]	0.31 (± 0.53) [0.0–2.3]	0.54 (± 0.73) [0.0–2.3]	0.25
On oral CAI (*n*, %)	6 (11%)	0 (0%)	6 (17%)	0.085
Previous non-glaucoma ocular surgery (%)	37 (69%)	14 (78%)	23 (64%)	0.36
Previous glaucoma surgery (%)				
Trabeculectomy	38 (70%)	13 (72%)	25 (69%)	1.00
Ahmed tube shunt	3 (6%)	1 (6%)	2 (6%)	1.00
Baerveldt tube shunt	21 (39%)	7 (39%)	14 (39%)	1.00
Others (SPI, canaloplasty, cypass/Xen)	5 (9%)	1 (6%)	4 (11%)	0.65
Number of previous glaucoma surgeries (*n*, ± SD, [range])	1.8 (± 0.6) [1–3]	1.6 (± 0.8) [1–3]	1.8 (± 0.6) [1–3]	0.23

*SD*, standard deviation; *IOP*, intraocular pressure; *CAI*, carbonic anhydrase inhibitor; *SPI*, surgical peripheral iridotomy

### Treatment

Of the 18 eyes with Xen stents, 17 implantations were performed stand-alone; 1 eye underwent combined Xen stent implantation with phacoemulsification and intraocular lens implantation. Of the 36 control eyes, 25 (69%) eyes underwent GDD implantation (24 Baerveldt [Johnson & Johnson], 1 Ahmed FP7 [New World Medical]) and 11 (31%) trans-scleral continuous wave CPC. One of 18 Xen eyes and six of the 36 control eyes underwent combined phacoemulsification with intraocular lens implantation with the glaucoma surgery (*P* = 0.40). No intraoperative complications were observed in either group.

### Failure

A total of 5 Xen eyes and 8 control eyes failed within the follow-up period. Most eyes failed due to requiring additional glaucoma surgery, except 1 control eye that failed due to loss of light perception. Three of 5 Xen failure eyes failed within the first 6 months postoperatively.

**Fig. 1:**
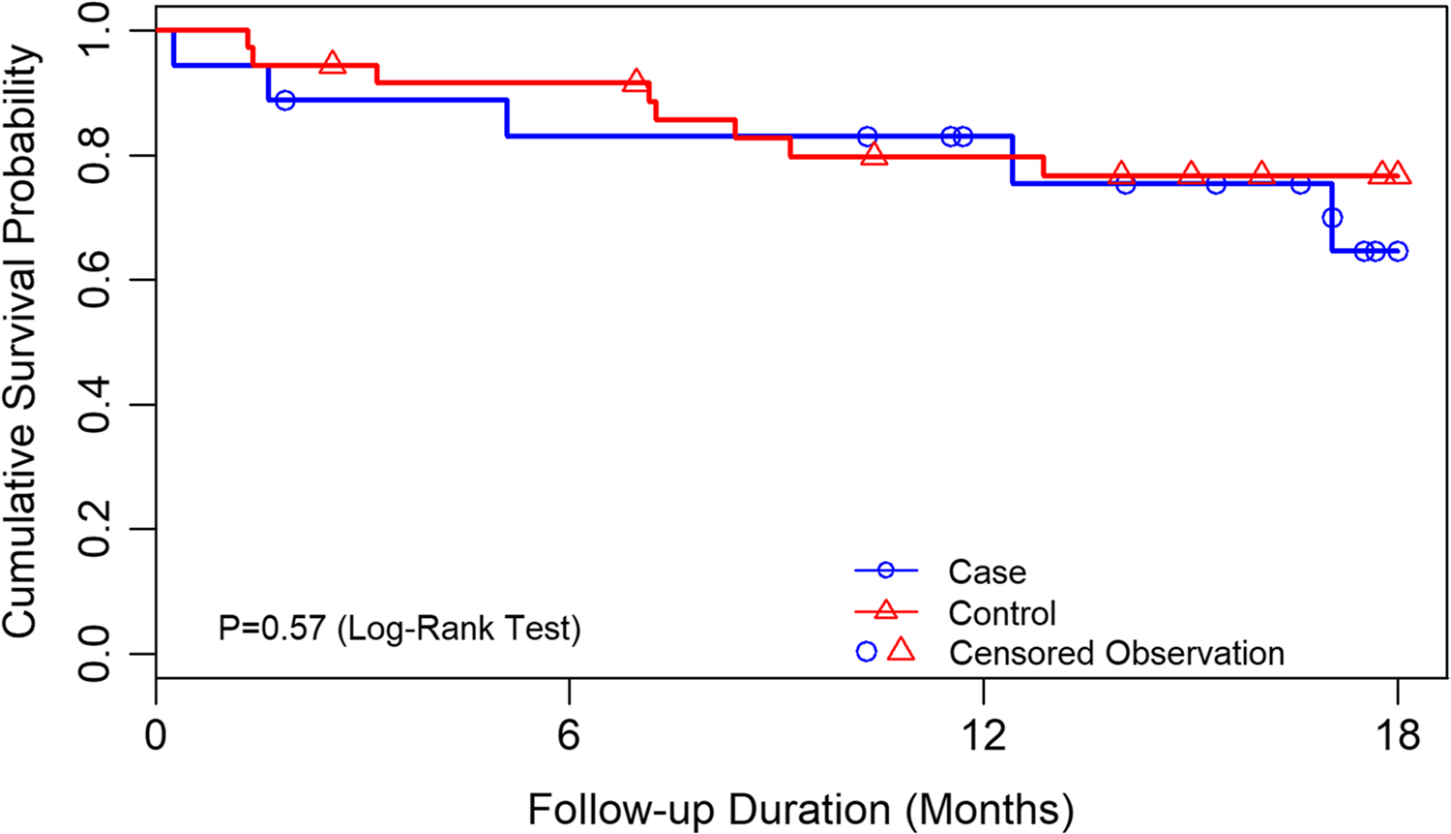
Kaplan–Meier survival curve. Failure was defined as meeting any of the following conditions in the follow-up period: (1) no light perception (NLP) vision; (2) additional glaucoma surgery required; (3) persistent IOP of less than 6 mm Hg after 6 weeks postoperatively

The cumulative failure rate at 1 year was 17% for the Xen group and 20% for the control group. Conversely, the success rate at year 1 was 83% for the Xen group and 80% for controls. The mean survival time was 14.1 (± 1.5) months and 11.4 (± 0.6) months for the Xen and control groups, respectively. Figure 1 shows the Kaplan–Meier survival curves. There was no significant difference between groups (*P* = 0.57, log-rank test).

### Additional procedures

In the 5 Xen eyes that failed due to requirement for further surgery, 6 further procedures were performed. Two eyes underwent CPC, 1 eye had a GDD placed, and 2 eyes had a second Xen stent placed. Of the 2 eyes that underwent a second Xen stent implantation, 1 eye underwent a GDD placement 3 months later.

Of the 7 control eyes which required additional glaucoma surgery, 6 eyes underwent CPC. One of the 6 had simultaneous GDD removal at the time of CPC, and one eye had GDD removal sequentially after CPC. The remaining eye had GDD removal with no additional glaucoma procedures due to infection. The rates of additional surgeries were not significantly different between the Xen and control groups (*P* = 0.41, Poisson regression).

### Complications

Two eyes in the Xen group experienced complications during follow-up (Table 2). One eye had a self-limited choroidal detachment which was considered a serious vision threatening complication. The other eye had a posteriorly malpositioned Xen that was found postoperatively; a second Xen stent was subsequently implanted but the first Xen placed was not surgically manipulated, thus this case was considered a surgical failure for analysis, with a minor complication.

**Table 2:** Postoperative complications

Complication	Xen (*N* = 18)	Control (*N* = 36)
Minor complications (*P* = 0.65)	**1**	**4**
Hyphema	0	4
Xen malposition	1	0
Serious complications (*P* = 0.043)	**1**	**12**
*Visual threatening*	*1*	*8*
Tube/Xen exposure	0	2
Blebitis	0	1
Cystoid macular edema	0	2
Choroidal effusion	1	2
Hemorrhagic choroidal detachment	0	1
Requiring surgical intervention in the operating room*	*0*	*4*
Iris plugging tube	0	2
Tube malposition	0	2
Total	2	16

^*^Not included in vision threatening complications but requiring surgical intervention

In the control group, there were 16 complications in 13 eyes (Table 2). The most frequently observed complication was hyphema (*n* = 4), followed by tube exposure, tube malposition, cystoid macular edema, choroidal effusion, and iris plugging the GDD tube (*n* = 2 each). The eyes with tube exposure (*n* = 2) and blebitis (*n* = 1) required tube explantation, as did 1 eye which had a malpositioned tube. All cases of choroidal effusion, hemorrhagic choroidal detachment, and hyphema were self-resolving.

There was no statistically significant difference in overall complication rates between groups (*P* = 0.098, Poisson regression) when adjusted for length of follow-up. Thirteen serious complications (1 in Xen and 12 in control) that were vision threatening or required additional surgery intervention in the operating room were observed in 1 Xen eye and 11 control eyes. The percent of eyes experiencing serious complications were significantly different between 2 groups (*P* = 0.043, Fisher exact test). One minor complication occurred in the Xen group while 4 minor complications occurred in 4 eyes within the control group (*P* = 0.65). Furthermore, both complications in the Xen group and 13 complications from 11 eyes in the control group occurred in the first 3 months postoperatively. The number of complications that occurred in the first 3 months postoperatively was not statistically different between the two groups (*P* = 0.12, Poisson regression).

### Revisions

There were 10 revisions in 10 Xen eyes; in the control group, there were 6 revisions in 6 eyes. All revisions in the Xen group were bleb needling procedures and occurred from 29 to 183 days postoperatively (mean 82.1 [± 48.8] days). All revisions in the control group were performed on GDDs and were either related to iris plugging the tube (*n* = 2) or tube malposition (*n* = 2). One revision occurred in a pars plana tube shunt and required pars plana vitrectomy to remove vitreous from the lumen. After adjusting for follow-up duration, the Xen group was 3.9 times more likely to require a revision (95% CI [1.4, 10.6]; *P* = 0.009, log-linear model).

### Intraocular pressure, IOP-lowering medications, and hypotony

Follow-up IOP measurements and number of IOP-lowering medications (censored at the time of additional glaucoma surgery) were not significantly different between groups (Table 3). Mean IOP was significantly reduced compared to baseline in both groups (*P* < 0.05), except for week 3 in the Xen group (*P* = 0.31). The mean IOP reduction from baseline was not significantly different between the groups, again except during week 3 (*P* = 0.020). Mean IOP dropped from 21.78 (± 7.24) to 15.31 mmHg (± 3.75), a 25% reduction, in the Xen group and from 27.09 (± 9.48) to 15.11 (± 5.12), a 40% reduction, in the control group at year 1 (*P* = 0.90) when censored for failures.

**Table 3:** Follow-up IOP and number of hypotensive medications censored at the time of additional glaucoma surgery

Visit	Xen		Control		*P*	Xen		Control		*P*
	*N*	Mean (± SD)	*N*	Mean (± SD)		*N*	Mean (± SD) [*P* value]	*N*	Mean (± SD) [*P* value]	
	IOP (mmHg)					IOP reduction from baseline (mmHg)				
Baseline	18	21.78 (± 7.24)	36	27.09 (± 9.48)	0.043		-	-	-	-
Day 1	17	7.53 (± 6.85)	25	15.80 (± 12.33)	0.008	17	− 13.82 (± 9.01) [*P* < 0.001]	25	− 13.12 (± 14.11) [*P* < 0.001]	0.86
Week 1	17	11.41 (± 6.24)	33	14.73 (± 10.68)	0.17	17	− 10.41 (± 9.49) [*P* < 0.001]	33	− 12.91 (± 10.65) [*P* < 0.001]	0.42
Week 3	16	17.81 (± 7.72)	35	16.11 (± 8.61)	0.50	16	− 2.63 (± 10.09) [*P* = 0.31]	35	− 10.97 (± 12.05) [*P* < 0.001]	0.020
Month 3	16	15.81 (± 5.67)	32	17.66 (± 6.66)	0.35	16	− 5.69 (± 9.32) [*P* = 0.027]	32	− 9.69 (± 10.36) [*P* < 0.001]	0.20
Month 6	14	15.79 (± 4.68)	30	16.70 (± 5.88)	0.61	14	− 5.93 (± 9.51) [*P* = 0.036]	29	− 10.52 (± 10.06) [*P* < 0.001]	0.16
Year 1	13	15.31 (± 3.75)	27	15.11 (± 5.12)	0.90	13	− 6.85 (± 7.63) [*P* = 0.007]	27	− 11.70 (± 9.13) [*P* < 0.001]	0.11
	Number of IOP-lowering medications					Number of IOP-lowering medication reduction from baseline				
Baseline	18	2.28 (± 1.18)	35	2.83 (± 1.07)	0.093	-	-	-	-	-
Day 1	18	0.11 (± 0.47)	25	0.12 (± 0.6)	0.96	18	− 2.17 (± 1.20) [*P* < 0.001]	25	− 2.76 (± 1.13) [*P* < 0.001]	0.11
Week 1	17	0.0 (± 0.0)	33	1.24 (± 2.11)	0.002	17	− 2.29 (± 1.21) [*P* < 0.001]	33	− 1.64 (± 2.19) [*P* < 0.001]	0.18
Week 3	16	0.06 (± 0.25)	35	0.86 (± 1.26)	0.001	16	− 2.25 (± 1.06) [*P* < 0.001]	35	− 2.03 (± 1.42) [*P* < 0.001]	0.58
Month 3	16	0.31 (± 0.70)	32	1.06 (± 1.13)	0.020	16	− 1.81 (± 1.11) [*P* < 0.001]	32	− 1.78 (± 1.21) [*P* < 0.001]	0.93
Month 6	14	0.86 (± 1.17)	30	1.47 (± 1.25)	0.13	14	− 1.36 (± 1.01) [*P* < 0.001]	29	− 1.41 (± 1.52) [*P* < 0.001]	0.90
Year 1	13	1.23 (± 1.17)	27	1.56 (± 1.09)	0.39	13	− 1.08 (± 1.19) [*P* = 0.007]	27	− 1.37 (± 1.36) [*P* < 0.001]	0.51

*IOP*, intraocular pressure

The mean number of IOP-lowering medications dropped from 2.28 (± 1.18) to 1.23 (± 1.17) in the Xen group and from 2.83 (± 1.07) to 1.56 (± 1.09) in the control group at year 1 postoperatively (*P* = 0.51). Although the mean number of IOP-lowering medications in the Xen group was significantly less than the control group at week 1 (*P* = 0.002), week 3 (*P* = 0.001), and month 3 (*P* = 0.020), the mean reduction in the number of IOP-lowering medications was not significantly different between groups (*P* ≥ 0.11 for all study time points).

All cases of hypotony (IOP < 6 mm Hg) were early and occurred within 3 weeks postoperatively. There were no cases of persistent hypotony or hypotony maculopathy. The incidence of early hypotony in the Xen group was 44% (*n* = 8) and 18.4% (*n* = 7) in the control group (*P* = 0.10). No cases of early hypotony required surgical intervention.

### Visual acuity

Table 4 summarizes BCVA. Baseline BCVA was 0.31 (± 0.53) logMAR (approximately 20/40 Snellen) in the Xen group and 0.54 (± 0.73) logMAR (approximately 20/80 Snellen) in the control group (*P* = 0.25). BCVA did not significantly change from baseline after week 3 (*P* ≥ 0.17, paired *t* test) in both groups. There was 1 eye (6%) in the Xen group and 4 eyes (11%) in the control group with permanent loss of 2 lines or more of BCVA (*P* = 0.65).

**Table 4:** Follow-up best corrected visual acuity

	Best corrected visual acuity					Best corrected visual acuity change from baseline				
Visit	Xen		Control		*P*	Xen		Control		*P*
	*N*	Mean (± SD)	*N*	Mean (± SD)		*N*	Mean (± SD) [*P* value]	*N*	Mean (± SD) [*P* value]	
Baseline	18	0.31 (± 0.53)	36	0.54 (± 0.73)	0.25	-	-	-	-	-
Day 1	18	0.50 (± 0.52)	25	0.93 (± 0.82)	0.052	18	0.18 (± 0.20) [*P* = 0.003]	25	0.30 (± 0.55) [*P* = 0.014]	0.33
Week 1	17	0.50 (± 0.56)	33	0.75 (± 0.76)	0.24	17	0.19 (± 0.26) [*P* = 0.010]	33	0.17 (± 0.54) [*P* = 0.076]	0.90
Week 3	17	0.26 (± 0.25)	34	0.68 (± 0.75)	0.006	17	0.06 (± 0.16) [*P* = 0.13]	34	0.11 (± 0.45) [*P* = 0.16]	0.55
Month 3	18	0.33 (± 0.53)	34	0.55 (± 0.69)	0.23	18	0.01 (± 0.14) [*P* = 0.75]	34	− 0.00 (± 0.60) [*P* = 0.97]	0.89
Month 6	17	0.38 (± 0.67)	32	0.40 (± 0.58)	0.91	17	0.06 (± 0.45) [*P* = 0.56]	32	− 0.09 (± 0.45) [*P* = 0.27]	0.26
Year 1	16	0.36 (± 0.61)	33	0.46 (± 0.76)	0.63	16	0.07 (± 0.31) [*P* = 0.40]	33	0.04 (± 0.39) [*P* = 0.63]	0.33

## Discussion

Patients who have already undergone and failed prior incisional glaucoma surgery pose a unique challenge to the glaucoma surgeon. The previously manipulated conjunctiva leads to higher rates of surgical failure [19, 20]. In addition, multiple medications are usually inadequate or not tolerated prior to additional surgical intervention. While the studies on MIGS procedures are numerous, very few address the use of MIGS in previously operated on eyes. The no-longer commercially available CyPass Micro-stent (Alcon) was shown to be effective in reducing mean IOP by 33.7% at 1 year postoperatively in a cohort of 22 eyes with prior trabeculectomy or aqueous shunt surgery [21]. With the trabectome, Mosaed et al. found a 20% reduction in IOP at 1 year postoperatively in patients with prior GDD implantation, but there was no decrease in medication burden [22]. With gonioscopy-assisted transluminal trabeculotomy (GATT), Grover et al. achieved a 60–70% success rate in POAG eyes with prior incisional surgery at 24 months postoperatively, but with a cumulative proportion of reoperation of 29% at 24 months [23].

However, all of these studies are limited in design as retrospective case series, lacking control groups. To date, the only study comparing conjunctival-sparing surgery to traditional surgical intervention comes from Murakami et al.**,** who compared endoscopic cyclophotocoagulation (ECP) to second GDD placement in eyes with a single failed GDD. They reported no significant difference in mean IOP or IOP-lowering medication usage at 6 and 12 months postoperatively [24]. Our study is the first to evaluate the safety and efficacy of *ab externo* Xen stent implantation in eyes with uncontrolled IOP after trabeculectomy and/or GDD compared to the current standard of care (GDD or CPC; Pubmed search July 15, 2022 using terms Xen and glaucoma).

### Power

Despite the actual sample size being small, it remains adequate to detect a 20% difference in failure rate at 80% power. Our original power calculation assumed a failure rate of 5% in the control group which was taken from the ASSISTS trial [18]. Although our 1-year failure rate was 20% in the control group, which is greater than our original assumption, the actual difference in failure rate in this study was only 3% (17% for Xen and 20% for controls), which is nowhere near the 20% we considered clinically significant a priori. In addition, our sample size is sufficiently large to detect a 0.8 standard deviation difference between groups in continuous variables, such as IOP, number of IOP lowering medications, BCVA, and their changes from the baseline, at 80% power. However, we should note our sample size can only detect a difference of 30% or more at 80% power when comparing incidences of complications, revisions, and additional glaucoma procedures between groups.

### Failure

Failure in our study was defined as advancement to no light perception, late hypotony, or need for further glaucoma surgery. We chose these criteria because they represent real-world success in most eyes needing second surgeries. We made the decision not to include a required IOP reduction or percent reduction as part of the failure criteria for several reasons. First, one of the reasons surgery is required in this patient population may be due to intolerance of topical or systemic agents; thus, for some patients, reducing medication burden rather than IOP reduction is the goal of surgery. Second, many patients with glaucoma progression are placed on additional IOP-lowering agents or oral carbonic anhydrase inhibitors (CAIs) as temporizing measures while awaiting surgery, and the preoperative IOP may not represent a sustainable IOP with medications. Additionally, because there was no medication washout period for these eyes, preoperative and postoperative IOPs compare significantly different medication loads (Table 3). Surgeon preference dictated re-introduction of medications as needed in both Xen and control eyes.

In this context, the Xen performs non-inferiorly to the current standard of care (GDD or CPC) (Fig. 1). Three of 5 Xen failures occurred within the first 6 months, and failure was due to needing additional surgery. We should note that the baseline medicated IOP was lower in the Xen group compared to controls (*P* = 0.043) and the use of oral carbonic anhydrase inhibitors trended toward significance, with higher use in the control groups (*P* = 0.085). The baseline visual field mean deviation (MD) and staging by ICD-10, however, were not significantly different between the groups. This represents an inherent patient selection bias of this retrospective review. It should be noted that in published studies of predictors of surgical failure or success, baseline IOP, visual field mean deviation, and glaucoma staging have not been found to be predictors of success in primary Xen implantation [25], but are significant in primary tube shunt surgery [26, 27]. In CPC, baseline IOP was found to be a significant predictor of failure in neovascular glaucoma [28]. It should be noted that these studies have variable definitions of success and failure, some of which include a maximum allowed IOP and others which only define failure as reoperation.

Despite this difference in starting IOP, final medicated IOP, change from baseline, and number of IOP-lowering medication use were not significantly different between groups at all time points except week 3 (Table 3). In addition, despite this difference, the overall percent reduction of IOP was not significantly different: 25% in the Xen group and 40% in the control group (*P* = 0.90). The final IOP achieved at year 1 in both groups likely represents the surgical floor reached by filtering surgery [2, 12–14, 18].

One advantage to Xen stent implantation, particularly the transconjunctival *ab externo* approach, is the minimal manipulation of conjunctiva. The small caliber of the Xen stent allows it to be placed in eyes that previously would have been poor candidates for any further procedures other than cyclodestruction. In our cohort, we were able to place a Xen in 3 eyes that had already undergone both trabeculectomy and GDD surgery. Placement of a Xen also does not prevent future surgical intervention should therapy need to be advanced. In our study, of the 5 Xen eyes requiring further intervention, 2 were able to have subsequent GDDs placed. In contrast, all eyes of the control group that required additional surgery went on to receive a cyclodestructive procedure. Thus, a surgeon can potentially use the Xen device as a bridge to future therapy without losing the ability to place a GDD in the future should the Xen stent be insufficient.

### Complications

The Xen group had a significantly lower rate of serious complications which threatened sight or required surgical intervention in the operating suite (Table 2). This is particularly important in eyes that have already failed prior surgery, as they are inherently at higher risk of complications due to their complexity. There were no instances of Xen stent exposure or blebitis in our study, which is an important observation in eyes that have undergone prior surgical conjunctival manipulation, including in some cases exposure to repeat MMC administration. On the other hand, complications in the control group were quite variable in severity and were noted from day 1 postoperatively to as late as 408 days postoperatively. Most complications were managed conservatively. However, the eyes which had tube exposure (*n* = 2), blebitis (*n* = 1), and 1 eye with tube malpositioning required tube explantation. This rate of explantation is quite high (4 of 25 GDDs, 16%) at 18 months postoperatively, highlighting the complexity of GDD placement in eyes that have already had prior trabeculectomy and/or GDD placement.

It should be noted that in our study bleb needling was not considered a surgical failure or complication and was performed as a routine minor room procedure. Ten of 18 Xen eyes required needling (56%), all within the first 3 months postoperatively. This was 3.9-fold the rate of revision for the control group (*P* = 0.009). Though the rate of needling varies widely between published studies, from 25 to 53% [6, 11, 14, 16], this relatively high rate is likely due to the altered subconjunctival anatomy and predisposition to scarring in these eyes with preexisting conjunctival surgery. No adverse events after needling occurred; however, careful counseling of patients on this rate of needling as well as close follow-up of patients in the extended postoperative period should be expected.

We should also highlight that there is variability in both BCVA and IOP during the early preoperative phases between the groups. The Xen group had significantly better BCVA at day 1 and week 3 postoperatively compared to the controls (Table 4), although the change from baseline BCVA was not significant at any time point between the groups, and the early significance was lost at subsequent follow-ups. Not captured in this data is the functional difference patients may experience with this early decrease in postoperative vision. The Xen group also shows greater variability in IOP throughout the postoperative course (Table 3), with a tendency toward early hypotony. The control group demonstrated more consistent IOP lowering throughout all postoperative visits (Table 3). The Xen group does utilize significantly fewer IOP medications during the first 3 months postoperatively, which may explain some of the IOP variability. Other causes of variability are expected consequences of surgical technique, including early ligation off the tube and use of a wick for early IOP control. Similarly, patients who underwent CPC were asked to remain on their hypotensive agents in the immediate postoperative period and tapered off as the laser effect was seen.

### Limitations

Although adequately powered, our study has several limitations, mainly relating to its retrospective, single-center nature and short follow-up. As previously mentioned, baseline IOP was significantly lower in the Xen group, representing an inherent selection bias in which surgery was offered to or selected by patients. Although the results show no difference in final IOP or change in IOP between the groups, surgeons may want to consider baseline intraocular pressure when counseling patients on available options. Future prospective studies which can better match cases and control on baseline intraocular pressure would be valuable, but may be difficult to execute in a timely fashion as there are limited patients in this scenario. Similarly, only one of 18 Xen eyes and six of the 36 control eyes underwent phacoemulsification with intraocular lens implantation combined with the glaucoma surgery, a factor which would ideally be removed in future studies.

In addition, an *ab externo* transconjunctival approach augmented with mitomycin-C was used, and it is unknown if these results can be applied to other surgical techniques of Xen implantation including *ab interno* or open conjunctival *ab externo* modifications. Xen implantation was augmented with mitomycin-C at a concentration of 0.2 mg/mL to 0.8 mg/mL of MMC, a variable which would ideally be standardized in future studies. Lastly, while our cohorts do include multiple glaucoma types and stages, the majority of patients had primary open-angle glaucoma, and thus this study may under-represent certain subtypes of glaucoma.

## Conclusions

Despite these study limitations, our report helps validate the Xen gel stent as a reasonable alternative to GDD or CPC in eyes that have failed prior traditional glaucoma surgery. The Xen stent matches more traditional therapies in survival, and when successful, it achieves a 25% reduction in medicated IOP at 1 year with a favorable serious complication rate. Studies of longer duration are needed to elucidate the longevity of Xen implants in these eyes, but even “short term” success of months to years delaying more invasive treatment is valuable. Thus, the Xen stent is a reasonable alternative to GDD and CPC and can be considered a step in therapeutic management in these difficult-to-manage eyes.

## Data Availability

The datasets used and/or analyzed during the current study are available from the corresponding author on reasonable request.

## Abbreviations

BCVA: Best-corrected visual acuity
CAI: Carbonic anhydrase inhibitor
CPC: Cyclophotocoagulation
CPT: Current procedural terminology
ECP: Endoscopic cyclophotocoagulation
GATT: Gonioscopy-assisted transluminal trabeculectomy
GDD: Glaucoma drainage device
IOP: Intraocular pressure
MIGS: Microinvasive glaucoma surgery
MMC: Mitomycin-C
NLP: No light perception
POAG: Primary open angle glaucoma
MD: Mean deviation
UTHealth: The University of Texas Health Science Center at Houston
